# Identification of Myalgic Encephalomyelitis/Chronic Fatigue Syndrome-associated DNA methylation patterns

**DOI:** 10.1371/journal.pone.0201066

**Published:** 2018-07-23

**Authors:** Malav S. Trivedi, Elisa Oltra, Leonor Sarria, Natasha Rose, Vladimir Beljanski, Mary Ann Fletcher, Nancy G. Klimas, Lubov Nathanson

**Affiliations:** 1 College of Pharmacy, Nova Southeastern University, Fort Lauderdale, United States of America; 2 School of Medicine and Dentistry, Catholic University of Valencia, Valencia, Spain; 3 Institute for Neuro Immune Medicine, Dr. Kiran C. Patel College of Osteopathic Medicine, Nova Southeastern University, Fort Lauderdale, United States of America; 4 Cell Therapy Institute, Dr. Kiran C. Patel College of Allopathic Medicine, Nova Southeastern University, Fort Lauderdale, United States of America; 5 Miami VAMC, Miami, Florida, United States of America; Beijing Cancer Hospital, CHINA

## Abstract

**Background:**

Myalgic Encephalomyelitis/Chronic Fatigue Syndrome (ME/CFS) is a complex condition involving multiple organ systems and characterized by persistent/relapsing debilitating fatigue, immune dysfunction, neurological problems, and other symptoms not curable for at least 6 months. Disruption of DNA methylation patterns has been tied to various immune and neurological diseases; however, its status in ME/CFS remains uncertain. Our study aimed at identifying changes in the DNA methylation patterns that associate with ME/CFS.

**Methods:**

We extracted genomic DNA from peripheral blood mononuclear cells from 13 ME/CFS study subjects and 12 healthy controls and measured global DNA methylation by ELISA-like method and site-specific methylation status using Illumina MethylationEPIC microarrays. Pyrosequencing validation included 33 ME/CFS cases and 31 controls from two geographically distant cohorts.

**Results:**

Global DNA methylation levels of ME/CFS cases were similar to those of controls. However, microarray-based approach allowed detection of 17,296 differentially methylated CpG sites in 6,368 genes across regulatory elements and within coding regions of genes. Analysis of DNA methylation in promoter regions revealed 307 differentially methylated promoters. Ingenuity pathway analysis indicated that genes associated with differentially methylated promoters participated in at least 15 different pathways mostly related to cell signaling with a strong immune component.

**Conclusions:**

This is the first study that has explored genome-wide epigenetic changes associated with ME/CFS using the advanced Illumina MethylationEPIC microarrays covering about 850,000 CpG sites in two geographically distant cohorts of ME/CFS cases and matched controls. Our results are aligned with previous studies that indicate a dysregulation of the immune system in ME/CFS. They also suggest a potential role of epigenetic de-regulation in the pathobiology of ME/CFS. We propose screening of larger cohorts of ME/CFS cases to determine the external validity of these epigenetic changes in order to implement them as possible diagnostic markers in clinical setting.

## 1. Introduction

Myalgic Encephalomyelitis/Chronic Fatigue Syndrome (ME/CFS) is a condition that is characterized by an abrupt or delayed onset of persistent/relapsing symptomatology including memory and other neurological problems, muscle and joint pain, gastrointestinal issues, hormonal imbalance, immune dysfunction and debilitating fatigue. Moreover, such symptoms are usually unresolved with bed rest and are severe enough to impair average daily activity below 50 percent of usual activity level, lasting for a period of at least six months [1].

While the mechanism of ME/CFS remains unclear and diagnostic methods exclusively rely on symptomatology presentation and exclusion of laboratory findings, research efforts have demonstrated that ME/CFS impacts the endocrine, neurological, immune and metabolic processes resulting in impaired physiological homeostasis [2–4].

Statistical studies estimate the prevalence of ME/CFS at 0.23 to 0.41 percent [5, 6] of the general population, with a female to male ratio of 6:1 [7]. With this prevalence, annual costs to the United States economy have been estimated at $9 billion in lost productivity and up to $24 billion in health care expenditures [8–10]. Therefore, it seems that ME/CFS not only impacts an individual’s overall well-being and quality of life, but it also has far reaching effects on the society and economy and constitutes a significant public health concern.

Currently, treatment of ME/CFS relies only on the management of symptomatology [11] and improvement in quality of life due to a lack of understanding of the mechanisms underpinning disease onset and progression, limiting treatment options to partial and/or temporary relief of symptoms [11]. While some advances have been made in identifying molecular changes associated with ME/CFS, its complexity and the involvement of multiple organ systems have hindered the exact causes of the disease [12]. An improved understanding of the key molecular mechanisms of ME/CFS and dysfunction within regulatory systems will translate into appropriate diagnostic methods and management of cases, providing more targeted approaches to treatment.

Disruption of epigenetic mechanisms is linked to various immune, neurological and endocrine diseases [13–15]. Furthermore, DNA methylation patterns were found to be altered in several diseases often reported as comorbid to ME/CFS such as fibromyalgia (FM) and irritable bowel syndrome (IBS) [16, 17]. With respect to ME/CFS, we are aware of only a few studies, which examined differences in DNA methylation patterns between ME/CFS cases and controls [18–20]. These studies used Illumina Human Methylation450 BeadChip microarrays, which allow to analyze over 450,000 methylation sites per sample at single-nucleotide resolution. Other two additional studies limited the analysis to specific gene promoter regions using a site-specific approach for measuring DNA methylation in ME/CFS cases [21, 22]. The recently released Illumina MethylationEPIC microarrays allow analysis of DNA methylation changes on over 850,000 CpG sites [23]. This additional coverage should facilitate uncovering additional changes in transcription regulation present in ME/CFS subjects. In addition, by validating DNA methylation patterns associating with ME/CFS in 64 participants from two geographically distant independent cohorts using pyrosequencing, we identified consensus CpG hypomethylated sites which could be used in future screenings of associations of these epigenetic changes in ME/CFS. Therefore, this study intended to validate and build upon the previous analysis of genome-wide DNA methylation changes in ME/CFS and extend such findings by utilizing larger coverage of CpG sites. Future validation studies in larger cohorts of ME/CFS cases are warranted to provide reliable epigenetic markers in ME/CFS.

## 2. Materials and methods

### 2.1. Sample collection and processing

#### 2.1.1. Cohort recruitment

ME/CFS cases and controls were recruited from two locations: the Miami/Fort Lauderdale (Florida) area and Valencia, Spain as part of larger biomarker-oriented studies. All subjects had comparable age and body mass index (BMI) (Table 1). Because females are affected by ME/CFS more than males (6:1 ratio) [7], we recruited only female participants to reduce potential confounding effects. For inclusion we utilized the Fukuda [24] and Canadian [25] case definitions. Fukuda requiring fatigue of greater than 6 months duration and 4 of 8 symptom criteria including exercise induced relapse, myalgia, arthralgia, headache of a new and different type, non-restorative sleep, cognitive complaints, sore throat and tender lymph nodes [24], and Canadian requiring exercise induced relapse and symptoms form at least of 3 categories (immune, autonomic, endocrine) [25].

**Table 1:** Demographic information and SF-36 results for all ME/CFS cases (n = 33) and controls (n = 31) that participated in DNA methylation analysis and validation. *—p<0.05, Student’s t-test, ME/CFS cases versus controls subjects. Data are shown as mean ± standard error of mean.

		ME/CFS Cases	Healthy Controls
	**Age (years)**	**49.9 ± 1.56**	**47.9 ± 1.63**
	**BMI (kg/m**^**2**^**)**	**25.8 ± 0.90**	**25.6 ± 0.80**
**Physical Health**			
	**Physical Functioning**	39.3 ± 4.69*	**96.8 ± 0.91**
	**Role Physical**	18.5 ± 7.01*	**91.7 ± 4.69**
	**Bodily Pain**	32.6 ± 4.86*	**89.1 ± 3.12**
	**General Health**	26.9 ± 3.74*	**76.2 ± 3.55**
**Mental Health**			
	**Vitality**	22.4 ± 4.40*	**71.2 ± 3.94**
	**Social Functioning**	34.5 ± 5.52*	**92.2 ± 2.56**
	**Role Emotional**	49.4 ± 8.42*	**87.8 ± 5.42**
	**Mental Health**	60.7 ± 4.83*	**79.6 ± 3.52**

All ME/CFS study subjects had the Medical Outcomes Study 36-item short-form survey (SF-36) summary physical score below the 50th percentile, based on population norms. All subjects were between 30 and 60 years old when samples were collected.

Exclusion criteria: all ME/CFS subjects, selected by their medical history and physical examination, had no history of malignancy or other systemic disorders including the listed psychiatric exclusions required for a diagnosis of ME/CFS, as clarified by Reeves [26], using the Composite International Diagnostic Instrument (a computerized interview format assessment of comorbid or exclusionary psychiatric diagnoses) [27]. Additional exclusion criteria included: active smoking or alcohol history, taking medications that could potentially impact immune function (e.g. steroids, immune-suppressors, etc.), taking diuretics, beta blockers, calcium channel blockers, ACE inhibitors, as well as subjects with a BMI > 35. Controls were self-defined as healthy, sedentary (no regular exercise program, sedentary employment), and matched to ME/CFS cases by age (+/- 5 years), race/ethnicity and BMI (+/- 5). All subjects were asked to complete the Gynecologic Questionnaire to assess routine gynecologic parameters and were asked to come for the assessment and collection of blood during the first two weeks of their menstrual cycle if premenopausal.

All subjects signed an informed consent approved by the Institutional Review Board of the Nova Southeastern University. Ethics review and approval for the subjects from Valencia was obtained from Hospital de La Plana (Villarreal, Spain) Ethics Committee. All subjects from Valencia (Spain) signed an informed consent before they could be included in the study.

The following questionnaires were completed for all enrolled subjects (cases and controls): the Multidimensional Fatigue Inventory (MFI) [28] to measure fatigue, the SF-36 [29] to assess health-related quality of life, the Krupp Fatigue Severity Inventory (Krupp FSI) [30] to measure perception of fatigue severity, the Sickness Impact Profile [31] to measure the impact of symptoms on the activities of daily life.

Student t-test was used to assess statistical difference between ME/CFS cases and controls in SF-36 scores for physical and mental health.

#### 2.1.3. Isolation of PBMCs and DNA extractions

Blood samples (8 ml) collected and kept at room temperature in K_2_EDTA (Becton Dickinson) were processed within 2 h by dilution at 1:1 (v/v) ratio in phosphate-buffered saline solution (PBS), layering on top of 1 volume of Ficoll-Paque Premium (GE Healthcare) and separation by density centrifugation at 20°C at 500 g for 30 min (brakes off). The PBMC layer was isolated and washed with PBS. The isolated PBMC pellets were resuspended in 1 volume of red blood cell lysis buffer (155 mM NH_4_Cl, 10 mM NaHCO_3_, pH 7.4, and 0.1 mM EDTA), kept on ice for 5 min, and centrifuged (20°C, 500 g, 10 min). The washed pellets were resuspended in freezing medium (90% FBS, 10% DMSO) adjusting their concentration to 10^7^ cells/ml, aliquoted and frozen at -150°C or liquid nitrogen until use. Genomic DNA was extracted from rapidly thawed PBMCs using DNeasy Blood & Tissue Kit (Qiagen), according to manufacturer´s instructions. In brief, pelleted PBMCs were resuspended in 200 μl of PBS supplemented with proteinase K and RNase A and genomic DNA was obtained from treated lysates using the kit binding columns and binding/wash/elution standard conditions. DNA quality and concentration were assessed by Agilent TapeStation 4200 (Agilent Technologies). All DNA samples had DNA Integrity Number (DIN) above 8 (data not shown).

### 2.2. Genomic DNA methylation profiling

For global epigenetic measurements MethylFlash Methylated DNA 5-mC Quantification Kit (EpiGentek) and MethylFlash Methylated DNA 5-hmC Quantification Kit (EpiGentek) have been used with 100 ng of genomic DNA from each sample. DNA methylation and DNA hydroxy-methylation were quantified using 5-methylcytosine and 5-hydroxymetylcytosine respectively by an enzyme-linked immunosorbent assay-like reaction as previously described [32, 33]. The levels of methylated DNA were calculated using the absorbance on a microplate reader at 450 nm. Results were normalized against a standard curve prepared according the manufacturer’s protocol using reference 0–100% methylated standards.

For genome-wide DNA methylation assessment 500 ng of genomic DNA from each sample was submitted to the Center of Genome Technology of the John P. Hussman Institute for Human Genomics in the Miller School of Medicine University of Miami. Genomic DNA was bisulfite converted using the EZ DNA Methylation Kit (Zymo Research), and after processing, according to Illumina’s specifications, DNA was hybridized to Illumina MethylationEPIC microarrays [34].

### 2.3. Analysis of genomic DNA methylation data

Signal intensity data (IDAT files) were imported into R/Bioconductor (v.3.3.1) package RnBeads (v.1.2.0) [35]. Methylation values for each of the probes on the Illumina MethylationEPIC microarray were produced as beta-values, calculated as the ratio of methylated probe intensity over total intensity (methylated and unmethylated) for each probe, which range from 0 to 1, roughly corresponding to the methylation percentage of the probe [35]. Low-quality data (probes with detection p-value > 0.01) were removed from the analysis. Probes outside of the context (non-CpG) and invariable through all sample probes were filtered out. Probes overlapping SNPs were removed using RnBeads function “rnb.execute.snp.removal” with the option “snp =“any””. The resulting data set (649,836 probes) was normalized using BMIQ normalization of RnBeads package [36] and used for exploratory and differential methylation analysis. Differential methylation analysis was performed by using two strategies: genomic region-based and site-based. While the genomic region-based approach looks for the average methylation level of the genomic region (CpG islands, promoters, genes, and genome-wide tiling regions) and then compares the methylation status of the regions across samples, site-based approach analyses the CpG loci are analyzed one at a time. To detect regions or loci that are differentially methylated in ME/CFS cases compared to controls we used R/Bioconductor limma package [37] incorporated into RnBeads analysis. CpG sites were considered to be differentially methylated if they met the following selection criteria: the absolute beta-value difference between the mean beta-values of cases and controls was greater than 0.05, and false discovery rate (FDR) ≤ 0.05 using the Benjamini-Hochberg procedure [38] for microarray-based analysis and p≤ 0.05 according to the Student t-test for pyrosequencing-based analysis. Promoter regions (1500 bp upstream of the transcription start site (TSS) and 500 bp downstream of TSS) were considered to be differentially methylated if they met the following criteria: FDR ≤ 0.1 according to Benjamini-Hochberg procedure [38] and the absolute beta-value difference between the mean beta-values of cases and controls was greater than 0.05. RnBeads calculates the combined rank score for differential DNA methylation to each genomic region. This combined rank is defined as the maximum (worst) of three individual rankings of 1) by absolute difference in mean DNA methylation levels in the genomic region, 2) by the relative difference in mean DNA methylation levels, which is calculated as the absolute value of the logarithm of the quotient of mean DNA methylation levels, and 3) by the CpG-based or genomic region-based p-value that is combined from CpG-specific p-values of the region using an extension of Fisher’s method. We present our data in the Supplementary Tables ordered by the combined rank.

The raw microarray data have been deposited in Gene Expression Omnibus (GEO) at NCBI (https://www.ncbi.nlm.nih.gov/geo/) under the accession number GSE111183.

### 2.4. Validation of differentially methylated promoters by pyrosequencing

For validation of the data obtained with the Illumina MethylationEPIC microarrays by pyrosequencing, genomic DNA was treated with EZ DNA Methylation-Gold Kit (Zymo Research) according to the manufacturer’s protocol. Methylation of DNA was quantified with bisulfite treatment of DNA and simultaneous PCR. A 50-μl PCR was performed in 25 μl of GoTaq Green Master mix (Promega), with 1 pmol of biotinylated forward primer, 1 pmol of reverse primer and 50 ng of bisulfite treated genomic DNA. The primer sequences for TABPB were: forward—5’- GTGGATTTTGGGTGGGGATTA-3’ and reverse—5’- AACTCCCACCAAACCATCCTTACC-3’; for ZBTB18 were: forward—5’- GAGTTTGAGGAGATGTATTTGATATT-3’ and reverse—5’- AACTTTTCAACCAATTTATAAATCTTTTCT-3’. The PCR was followed by pyrosequencing, using conditions previously described [33]. Results were reported as the percentage of the 5-methylated cytosines with respect to total, methylated and unmethylated, cytosines. Additionally, non-CpG cytosine residues were used as internal controls to validate bisulfite conversion.

### 2.5. Ingenuity pathway analysis

Ingenuity Pathway Analysis software (IPA, Qiagen) was used to annotate genes with differentially methylated promoters (DMPs) and rank associated canonical pathways. Canonical pathways analysis identified the pathways from the IPA library of pathways that were most significant to the set of genes with DMPs. The significance of the association between a gene set and a canonical pathway was measured by determining the ratio between the number of genes in our list that mapped to the canonical pathway divided by the total number of all known genes that mapped to that pathway. Fisher’s exact test was used to calculate p‐values determining the probability that the association between the genes in the dataset and the canonical pathway was explained by chance alone.

## 3. Results

### 3.1. Study design and participant demographics

**Fig 1:**
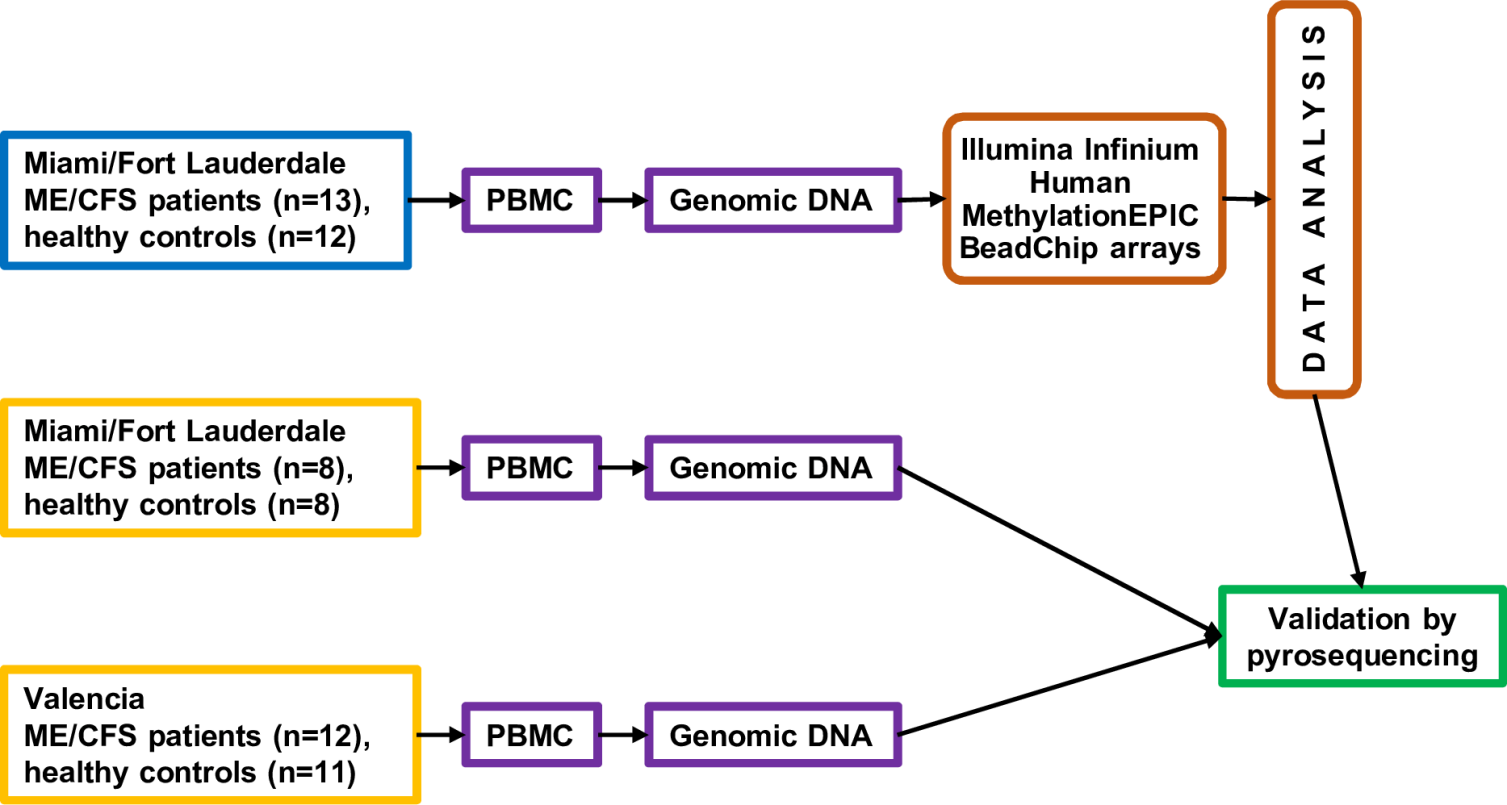
Study design flowchart. Genomic DNA was isolated from ME/CFS cases (N = 13) and controls (N = 12) and analyzed using Illumina Infinium Human MethylationEPIC BeadChip microarrays and this was defined as “experimental cohort”. Following data analysis results were validated on all the samples from the “experimental”, as well as from a secondary cohort that included samples from ME/CFS cases (N = 8) and controls (N = 8) from Miami/Fort Lauderdale area, as well as ME/CFS cases (N = 12) and controls (N = 11) from Valencia (Spain), using pyrosequencing analysis.

Flow chart of the study design is depicted on Fig 1. In brief, genomic DNA from 13 ME/CFS cases and 12 controls (“experimental cohort”) was hybridized to the Illumina MethylationEPIC microarrays, as detailed in Methods. These arrays can assay over 850,000 CpG sites and cover different regions across genes, including promoter, 5’ UTR, first exon, gene body, and 3’ UTR, as well as and distant regions. Coverage includes 99% of RefSeq genes with multiple probes per gene, 96% of CpG islands from the UCSC (University of California, Santa Cruz) database and additional content imputed from whole-genome bisulfite sequencing data [23]. Following data analysis, the obtained results were validated by pyrosequencing on the samples from the experimental cohort, as well as on additional 8 ME/CFS cases and 8 controls from Miami/Fort Lauderdale area and 12 ME/CFS cases and 11 controls from Valencia (Spain), as described in Methods.

It is worth noting that previous studies have had similar number of cases and controls using different Illumina Human Methylation450 microarrays. For example, Brenu et al [20] included 25 ME/CFS cases with 18 controls and measured methylation in T-cells; whereas De Vega et al [18] included 12 ME/CFS cases and 12 controls and measured methylation status in PBMCs. Hence, our sample size is similar to these previous publications for statistical comparison. Moreover, the major strength of the current findings is depicted in the validation of specific CpG sites in an expanded cohort of ME/CFS cases and controls from a different geographical location i.e. Valencia, Spain. However, additional validation in a larger cohort is warranted.

Table 1 shows age and BMI matching of all ME/CFS cases and controls in the experimental cohort and in the 39 additional samples from two distant geographic locations (used for pyrosequencing validation). Vitality, social functioning, role emotional and mental health were significantly lower in ME/CFS cases compared to controls. In addition, statistically significant differences in physical health were found when cases were compared to controls, according to SF-36 scores. S1 and S2 Tables show age, BMI and SF-36 scores for the two separate cohorts, one from Florida, USA, and the other from Valencia, Spain, correspondingly.

### 3.2. Global methylation in ME/CFS

We did not find statistically significant differences in global DNA methylation levels using ELISA-based assay methods to detect either 5-methylcytosine (5-mC) or 5-hydroxymethylcytosine (5-hmC) modifications on DNA isolated from PBMCs of ME/CFS cases and controls (S3 Table). This analysis, however, shows overall cytosine modifications but it is limited at detecting specific fluctuations at particular locations in the genome.

Since former studies had shown site specific methylation differences in ME/CFS [18–21], we proceeded to evaluate this possibility in our experimental cohort, however, we used an upgraded version of the microarrays for human genome-wide methylation studies (Illumina Infinium Human MethylationEPIC microarrays). This novel technology which has almost twice coverage compared to that of previous Illumina HumanMethylation450 microarrays [36] may allow for validation and additions to previous findings in independent, geographically distant, cohorts of ME/CFS cases.

### 3.3. Differentially methylated CpG sites in ME/CFS

Data from the microarray-based genome-wide methylation analysis of the experimental cohort showed hypomethylation of the differentially methylated sites (DMS) (98%) in PBMCs from ME/CFS cases compared to controls.

**Fig 2:**
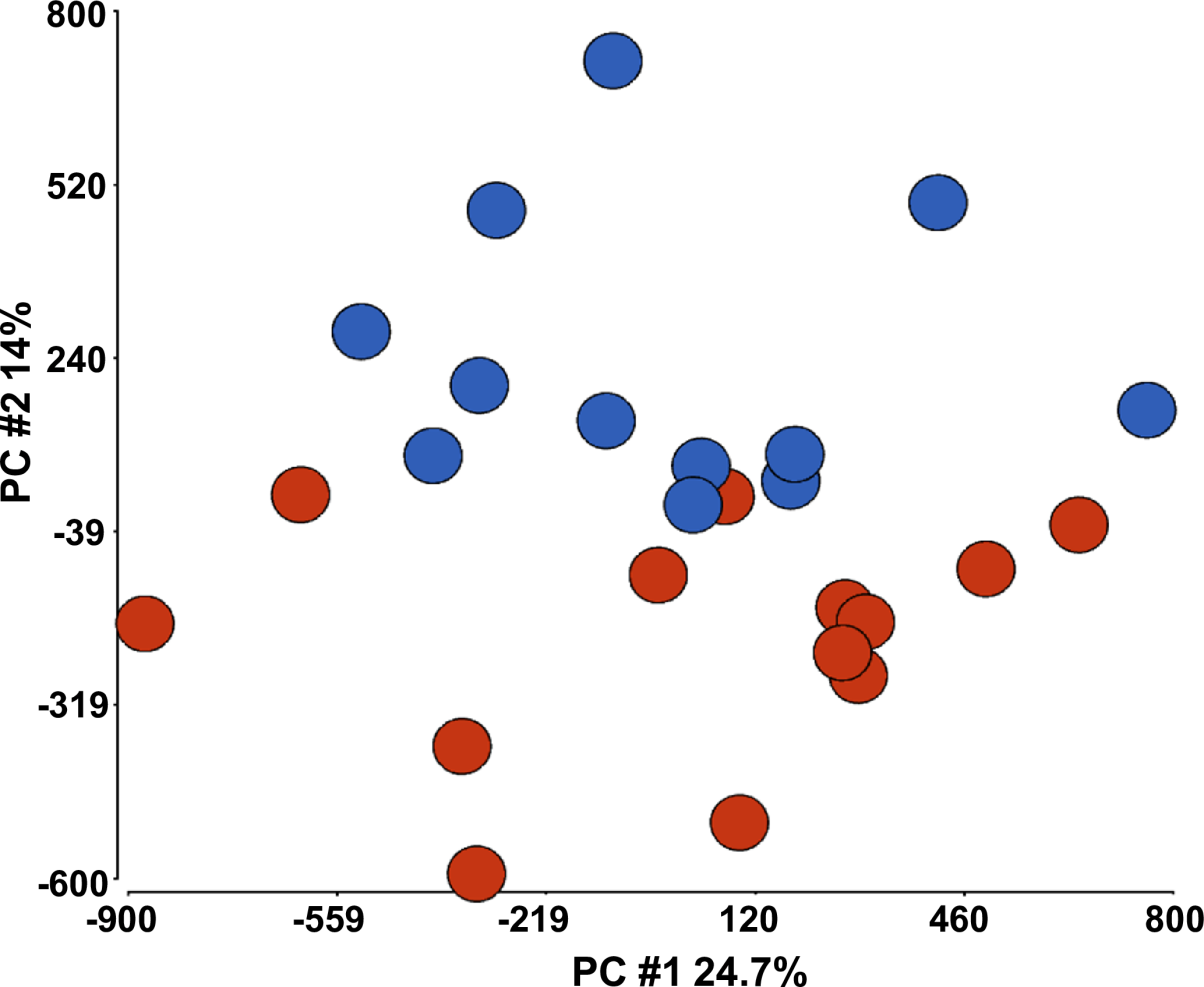
Principal component analysis of whole DNA methylomes allows differentiating between ME/CFS cases (N = 13, red circles) and matched controls (N = 12, blue circles).

**Fig 3:**
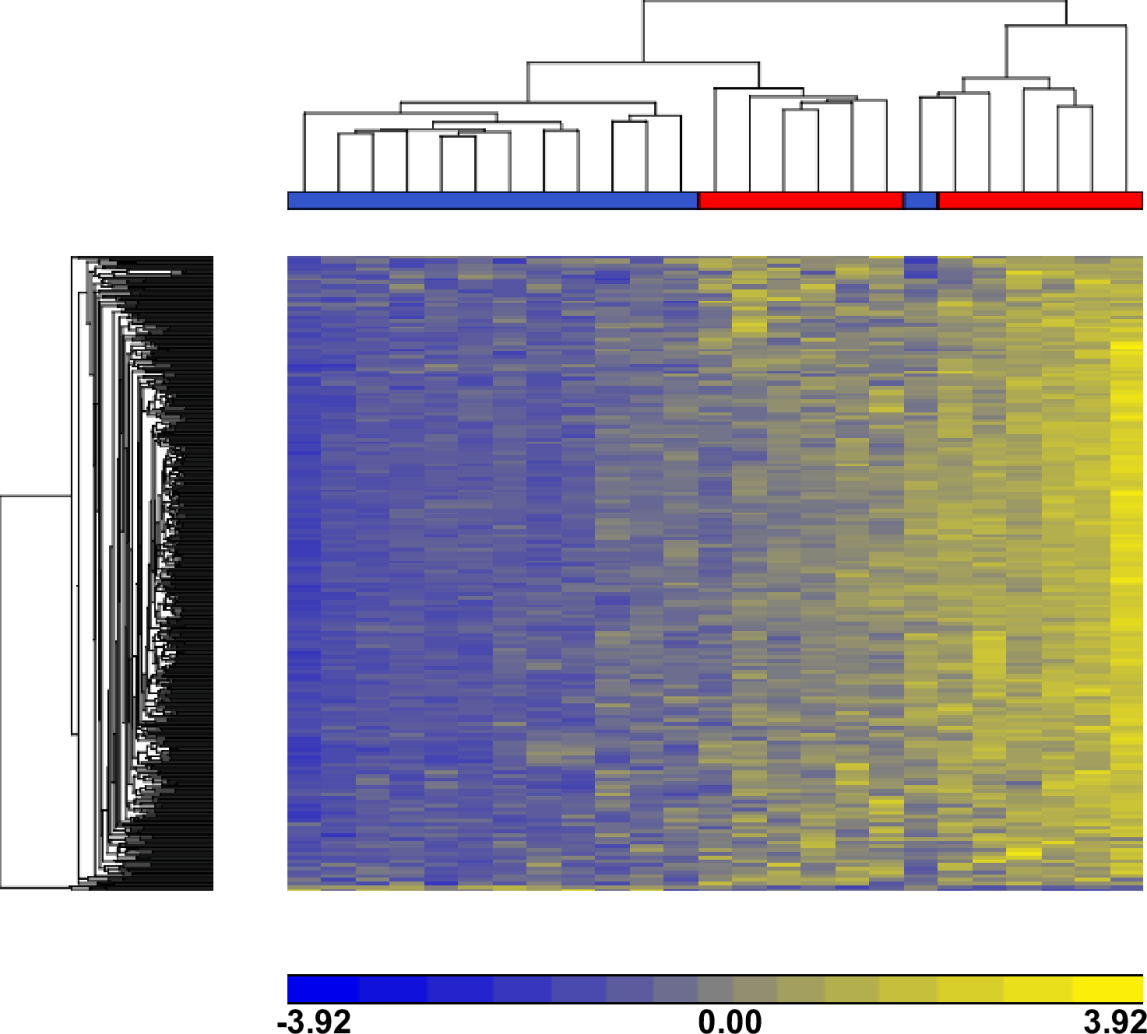
Unsupervised hierarchical clustering of 500 most variable CpG sites derived from samples distinguishes ME/CFS (N = 13) cases and controls (N = 12). 500 DMS above and below mean level are represented by blue and yellow across all cases and were used to cluster cases. As shown above, 12 of 13 ME/CFS cases (blue on color-coded bar above the dendrogram) cluster together (left dendrogram branch) and 12 of the 12 controls (blue color-coded bar) cluster together (right dendrogram branch), resulting in a divergence of these sub-phenotypes.

As many as 649,836 probes remained for normalization and further analysis upon filtering and preprocessing of the raw genomic DNA methylation data with the R/Bioconductor v.3.3.1 RnBeads package v.1.2.0. Analysis of these probes led to the finding of 17,296 DMS located in 6,368 annotated genes indicating epigenetic differences between the ME/CFS cases and controls in experimental cohort when we used FDR ≤ 0.05 criteria. S4 Table shows the list of DMS along with their respective gene references. Furthermore, Principal Component Analysis (PCA) of full methylomes clearly differentiated between ME/CFS cases and controls as shown in Fig 2. In addition, unsupervised hierarchical clustering of the 500 most variable CpG sites (according to RnBeads ranking, see Methods) shows clear segregation between ME/CFS cases and controls (Fig 3) confirming that methylation DNA patterns of PBMCs in ME/CFS individuals differ from those of controls.

**Fig 4:**
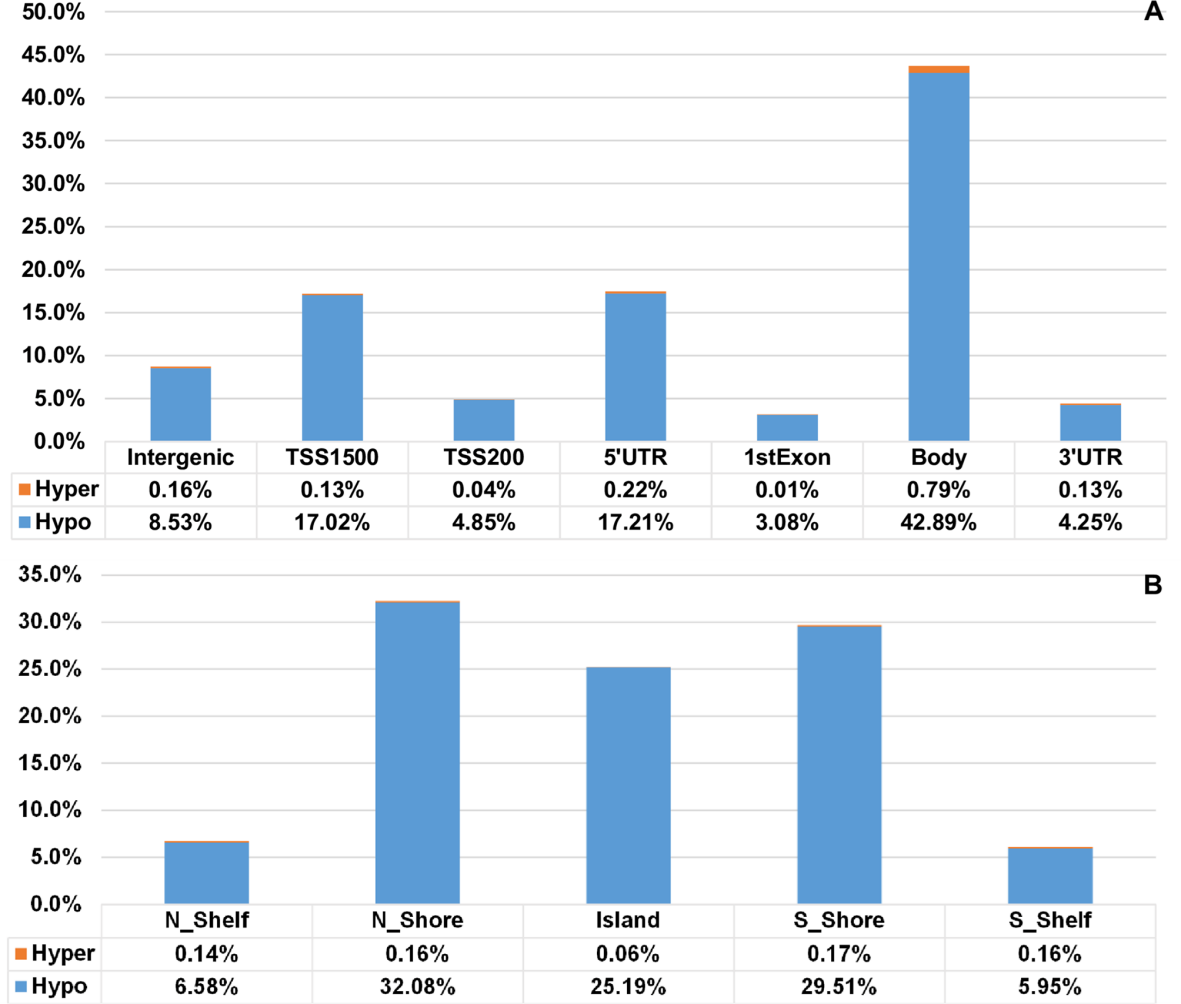
Distribution of differentially methylated sites according to genic location and to CpG island relation. (A) Distribution of DMS according to genic location. Relative percentages of DMS (both hypo-methylated and hyper-methylated CpG sites between ME/CFS cases and controls) are reported for each genic body location: gene regulatory elements (regulatory: TSS1500, TSS200, 5’ UTR, 3’ UTR), the coding regions of genes (gene body), as well as all Intergenic and 1st Exon gene body regions. (B) Distribution of DMS according to their location in relation to CpG islands. Relative percentages of DMS (both hypo-methylated and hyper-methylated) are reported. CpG islands with clustered CpG sites; 2 kb upstream and downstream of CpG islands (N, S Shores); 2 kb upstream and downstream of CpG shores (N, S Shelves).

Out of the 17,296 DMS, a total of 14,261 DMS (82%) were found within or proximal to genes (i.e., genic locations) indicating that they may have a role in regulation of gene expression. About 98% of the DMS within the genic regions were hypomethylated and 2% were hypermethylated overall (Fig 4A and S4 Table). These DMS were mostly equally distributed between gene bodies (43.68%) and gene regulatory elements (combined TSS1500, TSS200, 5’ UTR and 3’ UTR) (43.84%). Hypomethylation of ME/CFS DNA was observed on about 21.87% of DMS located in TSS regions; 17.21% in 5’ UTRs; 3.08% in 1st exons and 4.25% in 3’ UTRs. Hypermethylation, on the other hand, appeared on 0.16% of DMS located in TSS regions, 0.34% in 5 and 3’ UTRs, and 0.79% in gene bodies (Fig 4A). Regarding to the location relation to CpG islands, out of 17,296 DMS, 46.8% were located in CpG islands, whereas 53.2% (8,089 DMS) belong to proximity of CpG islands and CpG islands. As shown in Fig 4B, the mapping of 8,089 DMS with respect to their location in CpG islands shows that 99.3% of these 8,089 DMS are hypomethylated and only 0.7% were hypermethylated. Fig 4B also shows that out of these 8,089 DMS, approximately 25.25% were located within CpG islands, 61.9% mapped 2 kb upstream and downstream of CpG islands (N, S Shores), and 12.8% of them localized to regions 2 kb upstream and downstream of CpG shores (N, S Shelves). Within these regions, 74.1% of shores and shelves were hypomethylated, while only 0.63% of CpG island shores and shelves were hypermethylated, consistent with the general finding of hypomethylation being associated to ME/CFS. More relaxed criteria (FDR ≤ 0.1 and mean beta differences > 0.05) allowed to identify 27,129 DMS as listed in S5 Table.

### 3.4. Differentially methylated promoters in ME/CFS

**Fig 5:**
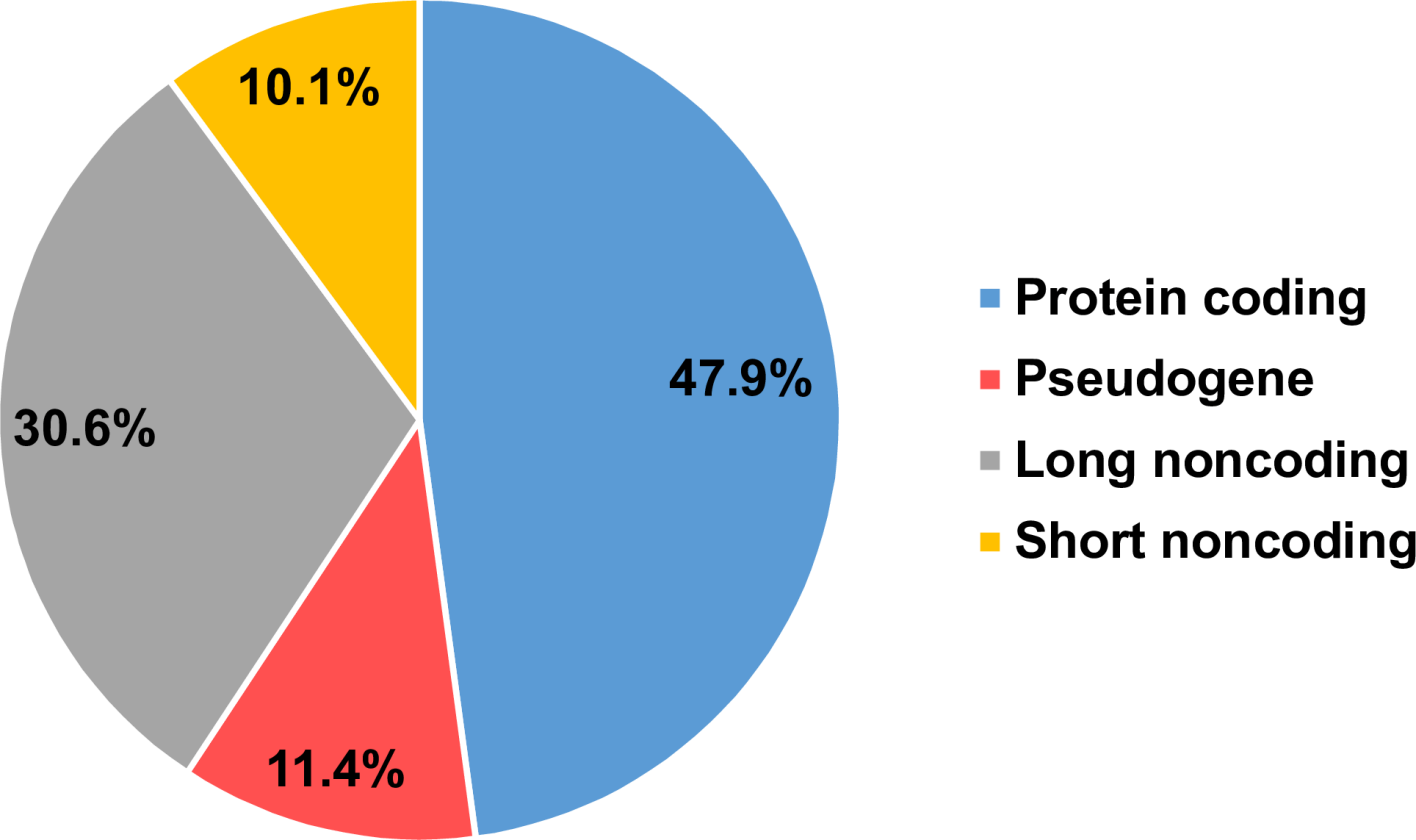
Distribution of differentially methylated promoter regions in ME/CFS according to gene biotype. In RnBeads package, promoters are defined as regions 1500 bp upstream of TSS and 500 bp downstream of TSS. Criteria for DMPs were: FDR ≤ 0.1 and mean difference > 0.05. The distribution of these DMPs according to the gene biotype: protein-coding genes and regulatory RNA genes including short non-coding RNAs (miRNAs, small nuclear RNAs, small nucleolar RNAs), long non-coding RNAs (including antisense RNAs and intergenic RNAs) and pseudogenes.

Since methylation of promoter regions directly affect levels of gene expression, we proceeded to analyze differentially methylated promoters (DMPs) in our experimental cohort. As described in Methods, promoters were defined in RnBeads package as regions located between 1,500 bp upstream of TSS and 500 bp downstream of TSS. Criteria for DMPs were FDR ≤ 0.1 with the mean beta differences > 0.05. Using these criteria, we found 307 DMPs in PBMCs of ME/CFS cases. S6 Table lists these DMPs ranked according to the RnBeads package [35]. 306 of these DMPs are hypomethylated in the ME/CFS group while only one appeared hypermethylated. Fig 5 shows the distribution of these promoters according to their gene biotype. Almost half of the identified DMPs belong to protein-coding genes (47.6%), and more than half (52.1%) belong to regulatory RNA genes. The last consisted of short non-coding RNAs (including miRNAs, small nuclear RNAs, small nucleolar RNAs) (10.1%), long non-coding RNAs (including antisense RNAs and intergenic RNAs) (30.6%), and pseudogenes (11.4%). This distribution of DMPs supports our hypothesis that the differential methylation observed might lead to deregulation of gene expression in ME/CFS. We also identified 144 DMPs with more stringent criteria of FDR ≤ 0.05 with the mean beta differences > 0.05 as listed in S7 Table; and it represents similar gene-function patterns as DMPs identified with FDR ≤ 0.1.

### 3.5. Functional pathway analysis of DMPs

**Fig 6:**
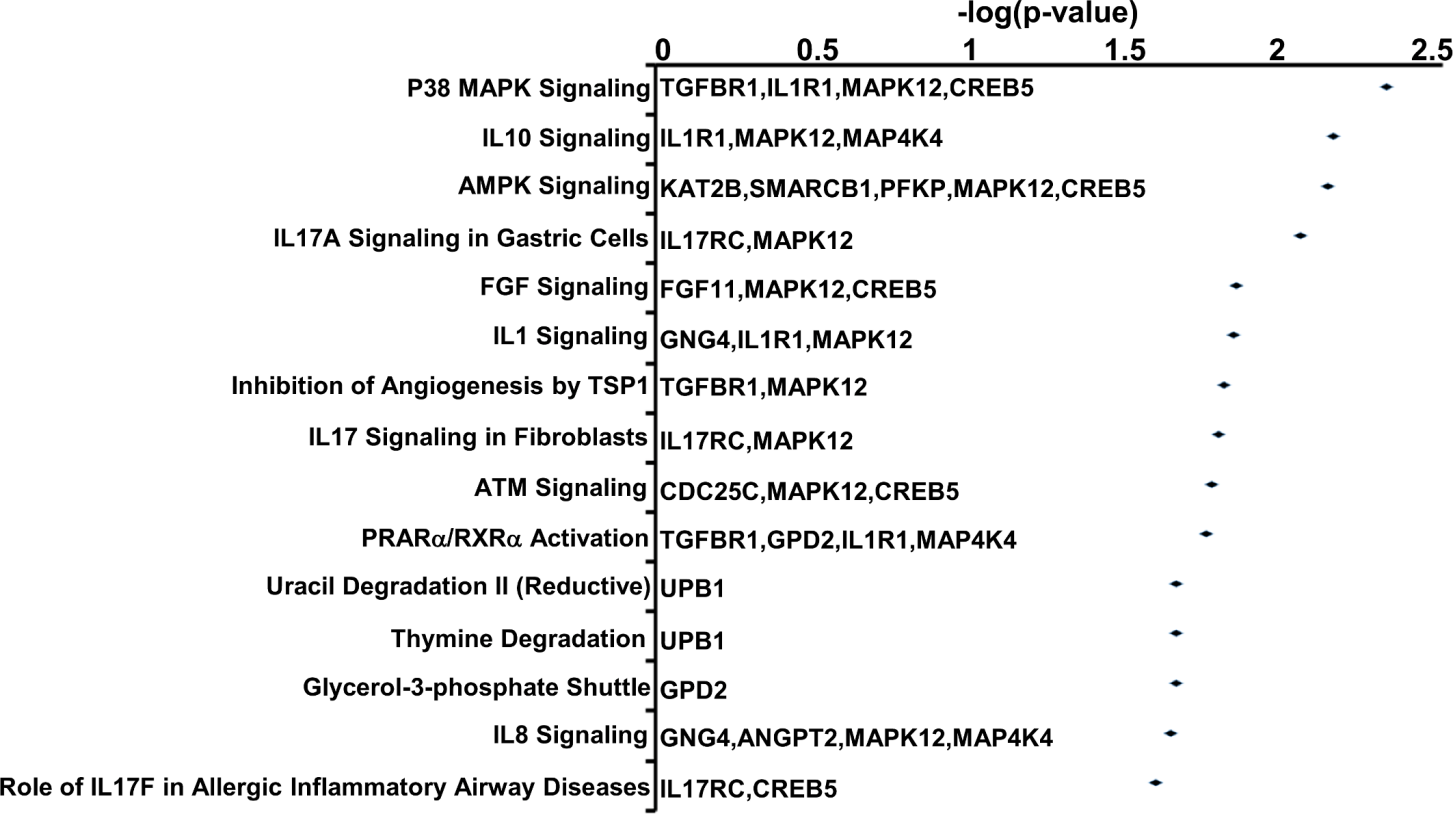
Canonical pathway analysis of genes with the DMPs of PBMCs isolated from ME/CFS cases. Genes listed to the right belong to the respective pathways. Gene Ontology analysis was performed using the Ingenuity Pathway Analysis (IPA) on 307 genes with the DMPs.

To further understand the potential functions and interactions between the 307 genes controlled by DMPs within the context of cell biological pathways, we performed Gene Ontology analysis through Ingenuity Pathway Analysis (IPA). As illustrated in Fig 6, this analysis revealed changes in numerous immunoregulatory/stress-response pathways such as the p38 MAPK pathway (involved in response to a variety of environmental stresses and to inflammatory cytokines [39]), the IL-10, IL-17A, Il-1 and IL-8 signaling pathways (all with important immunoregulatory roles, [40, 41]), the AMPK pathway (master regulator of cellular energy homeostasis that is activated in response to stresses that deplete cellular ATP supplies [42]) and metabolic pathways such as PPARα/RXRα activation and uracil and thymine degradation pathways [43, 44]. Furthermore, functional analysis of numerous individual genes revealed hypomethylation in promoters of the IGHJ1, IGHJ1P, IGHJ4, IGHJ5, IGHJ6, IGHJ3P, IGHV3-7, IGHD7-27, IGLL1 and LRMP genes (S5 Table) which are related to B cell immune response, differentiation, proliferation and survival [45]. Similarly, ME/CFS cases showed decreased DNA methylation level in promoters of genes responsible for T cell generation and activity, such as ZCCHC11, CAPN5, IL21R, P2RX5, TREML2, AKNA and CD248 [46]. Along with a robust impact in immune response pathways, PMBCs of ME/CFS cases showed decreased methylation of promoters of genes associated with cell growth that targeted the MAP Kinase, NF-kB, and TGF signaling pathways (S5 Table and Fig 6). In addition, a distinct pattern of hypomethylated promoters of genes involved in miRNA expression (miR-4435, miR-181, miR-148a, miR-193b, miR-1284, miR-10A, miR-1203, miR-3934, miR-4487 and miR-4710) and in bio-metal regulation including metal transporter SLC39A13 (ZIP transporter) and multiple zinc finger genes (ZCCHC8, ZCCHC11, ZBTB18, BAZ2B, ZNF225 and ZNF732) were identified (S5 Table).

### 3.6. Validation of Illumina MethylationEPIC microarray results by pyrosequencing

**Fig 7:**
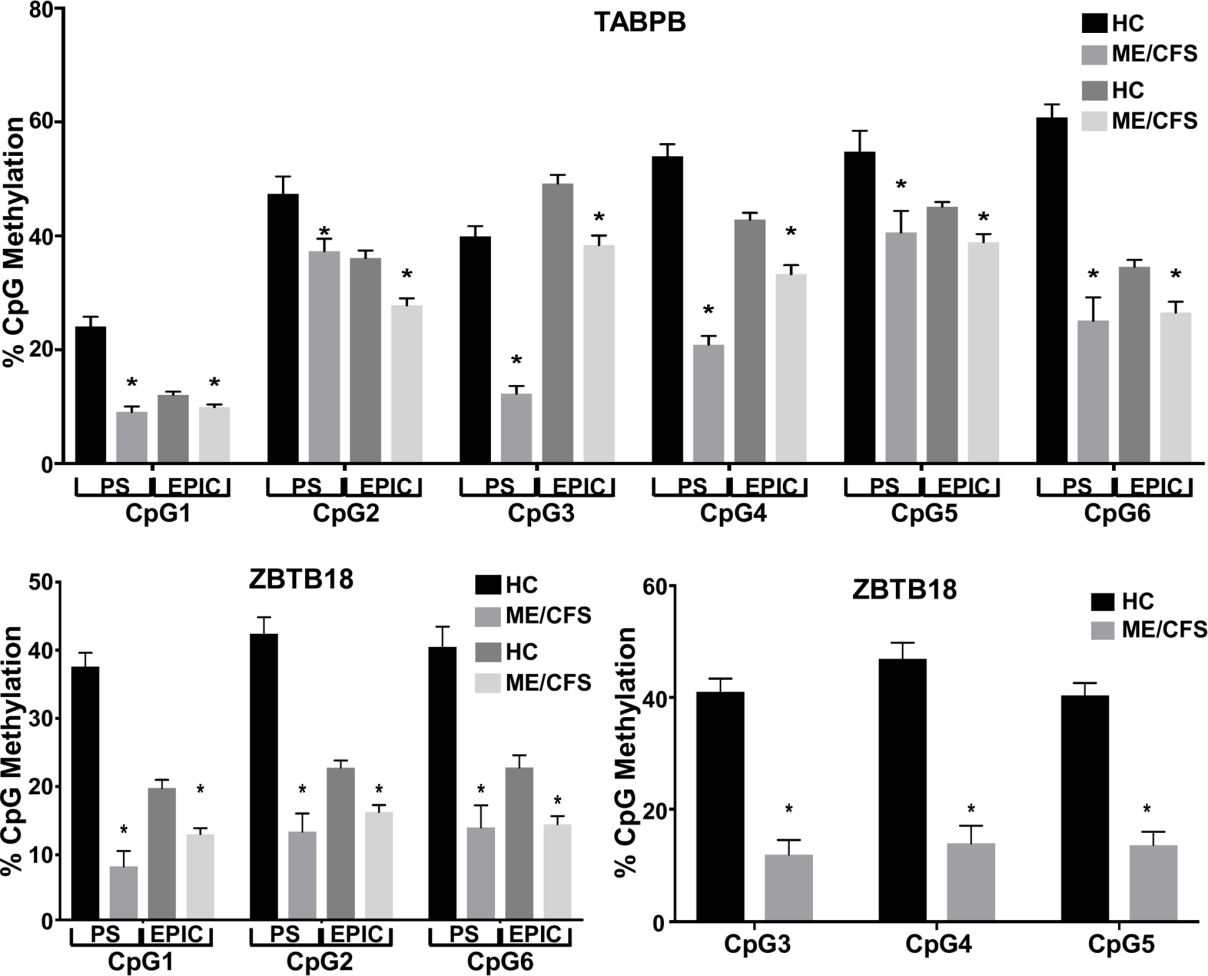
Bisulfite-conversion followed by pyrosequencing (PS) validation of DMPs identified by Illumina MethylationEPIC microarrays (EPIC). Average methylation level of CpG sites within promoters of TABPB and ZBTB18 were assessed using pyrosequencing. Corresponding probes for TABPB: CpG1—cg04415168 (TSS1500), CpG2—cg10376053 (TSS1500), CpG3—cg14288848 (TSS1500), CpG4—cg17055704 (TSS1500), CpG5—cg14473643 (TSS1500), CpG6—cg14309283 (TSS1500). Corresponding probes for ZBTB18: CpG1—cg16399365 (TSS1500), CpG2—cg15896892 (TSS1500), CpG6—cg19698993 (TSS1500). CpG3, CpG4 and CpG5 are not printed on the Illumina MethylationEPIC microarrays and are located in ZBTB18 promoter between CpG2 and CpG6. * = FDR ≤ 0.05 for EPIC; * = p ≤ 0.05 for PS. Error bars represent the standard error of the mean.

To validate the results of our analysis obtained with Illumina MethylationEPIC microarrays, we selected two DMPs that had high density of CpG sites and possible ME/CFS relevance by their role in the neuro-immune signaling axis. The first, corresponding to the Zinc Finger and BTB Domain Containing 18 (ZBTB18), is a zinc finger transcriptional repressor with a crucial role in brain development and neuronal differentiation. The second, TABPB, encodes a transmembrane glycoprotein which mediates interaction between newly assembled major histocompatibility complex (MHC) class I molecules and the transporter associated with antigen processing (TAP), which is required for the transport of antigenic peptides across the endoplasmic reticulum. ZBTB18 is in the upper top list of DMPs by order of RnBeads combined rank (position 8 of 307 in S5 Table); while TABPB is in the lower part (position 246 of 307 in S5 Table), thus we included DMPs with values at both ends of the range in the validation step. For each of the 2 selected genes, we assayed the level of DNA methylation of six CpG sites in the promoter area. For ZBTB18, three of the CpG sites corresponded to oligonucleotides printed on Illumina MethylationEPIC microarrays, and three additional CpG sites not included in microarray content but located in the gene promoter region between cg15896892 and cg19698993, were also tested. In the case of the TABPB gene, all the assayed CpG sites correlated with probes included in Illumina MethylationEPIC microarrays. As summarized in Fig 7, pyrosequencing analysis strongly validated the direction of methylation differences between ME/CFS and control subjects (p < 0.01) as identified by Illumina MethylationEPIC microarray (FDR ≤ 0.05) in all CpG sites assayed. Importantly, it was observed that the methylation levels detected by pyrosequencing in ME/CFS individuals were similar to those observed by MethylationEPIC microarrays in all DMS (Fig 7), and that the differences in methylation levels assayed by pyrosequencing were even more pronounced than the same differences assayed by microarrays (Fig 7).

## 4. Discussion

DNA methylation plays an important role in the interplay between external (environment) and internal (gene expression) factors and thus may explain the late or stimulus-triggered on-set of multisystem complex diseases such as ME/CFS. However, only a few studies have reported changes in DNA methylation associated to ME/CFS [18–20]. Here, we used a newer and improved, technology developed by Illumina Inc. that was not available at the time the preceding studies were performed, with the intention of expanding previous findings. Illumina MethylationEPIC microarrays have almost twice the coverage of CpG sites compared to the older Illumina HumanMethylation450 microarrays, including 333,265 additional CpGs located in regulatory regions as identified by the ENCODE and FANTOM5 projects [36].

Here, we identified differentially methylated CpG sites and promoters in PBMCs of ME/CFS cases compared to controls in cohorts from two distant geographic locations. Association of hypomethylation of DMPs with immune regulatory pathway is in agreement with previous studies [18–20]. Those previous studies found immune cell regulation as the largest coordinated enrichment of differentially methylated pathways and reported genomic DNA hypomethylation of genes in immune pathways from PBMCs isolated from ME/CFS cases [18, 19] and also in CD4+ T cells from ME/CFS cases [20]. Specifically, we found significant differences in DNA methylation between ME/CFS cases and controls at 17,296 CpG sites in 6,368 genes overall (FDR ≤ 0.05), across promoters as well as other gene regulatory elements and within coding regions of genes. DNA hypomethylation was found in 98% of these sites in ME/CFS, whereas only 2% were hypermethylated compared to controls.

Differential methylation of promoters can have an effect on the expression of the corresponding affected genes. Interestingly enough, less than half of the DMPs (47.6%) associated with protein-coding genes while over 50% affected non-protein coding DMPs corresponding to promoters of regulatory gene elements including short non-coding RNAs (including miRNAs, small nuclear RNAs, small nucleolar RNAs) (10.1%), long non-coding RNAs (including antisense RNAs and intergenic RNAs) (30.6%), and pseudogenes (11.4%) (Fig 5). Small nuclear RNAs and small nucleolar RNAs participate in RNA splicing [47] while long non-coding RNAs and pseudogenes play important roles in the regulation of transcription and mRNA stability by annealing to transcripts and sequestering miRNAs as molecular sponges [48], among other epigenetic mechanisms [49]. It is important to note that the highest beta difference (hypomethylation at 14%) in the DMP list was observed for the promoter of the gene that is related to natural killer (NK) cells function (S6 and S7 Tables).

At the single gene level, our results showed hypomethylation of promoters of genes related to those of reference studies such as some solute carriers, RNA binding proteins, homeobox, and PHD finger proteins [20]. Among them we found the MED13L gene which is associated with muscular hypotonia and neurocognitive impairment [50], symptoms that associate with ME/CFS. We also found DMPs linked to the expression of ribosomal proteins (RPL23A and RPL7) as well as to the 5S ribosomal RNAs pseudogenes (RNA5SP245, RNA5SP77 and RNA5SP97) (S5 Table) suggesting possible disturbance of protein synthesis. In regards to the methylation status of glucocorticoid response-associated genes, we found significant hypomethylation of the SGK3 (serum glucocorticoid regulated kinase) gene promoter [18, 19].

Transcriptional profiling studies in ME/CFS cases have pointed out perturbations in T-cell and B-cell activation and dysregulation in gene expression broadly related to immune responses [51–56], and such changes fit with other studies showing altered production of interleukin and interferon cytokines as features of ME/CFS immune dysfunction [57]. In the current study, we report hypomethylation of DMPs of genes known to regulate the adaptive immune response such as immunoglobulins and pseudogenes (for example, IGDCC3, IGHD7-27, IGHJ1, IGHJ3 and other) (S6 and S7 Tables) which is in agreement with the reported improvement of ME/CFS cases following B cell depletion therapy [58, 59]. We also observed hypomethylation of promoters of MMP14, MAP4K4, MAPK12 and CREB5 (S6 and S7 Tables) possibly activating the TNF signaling pathway, that fits with the reported over-expression of pro-inflammatory cytokines [60] in ME/CFS. In addition, we also show hypomethylation of miRNA-148a promoter that could potentially contribute to its overexpression. It is known that the miRNA-148-152 family can impair the innate immune response and antigen presentation of TLR-triggered dendritic cells [61]. Furthermore, miRNA-148a can contribute to DNA hypomethylation in lupus CD4+ T cells by repressing DNA methyltransferase 1 expression [62]. It is noteworthy to mention that amongst the DMPs associated to miRNA expression in this study (S5 Table), miRNA-10a, miRNA-181 and miRNA-4710 were also reported to be affected at the DNA methylation level in at least one previous ME/CFS study [20]. Specifically, miRNA-10a has been associated with ME/CFS in NK cells [63] and miR-181 is related to TLR-mediated inflammatory response [64], which is associated with ME/CFS [65].

Hypomethylation of the IL21R gene promoter (S6 and S7 Tables) may indicate increased IL21 receptor expression. The ligand binding of this receptor leads to the activation of multiple downstream signaling molecules, including JAK1, JAK3, STAT1, and STAT3. Knockout studies of the ortholog mouse counterpart suggest a role for this gene in regulating immunoglobulin production [66]. IL21 regulates both innate and adaptive immune responses, and also exerts major effects on inflammatory responses that promote the development of autoimmune diseases and inflammatory disorders [67]. Importantly, IL21 signaling is critical for induction of spontaneous experimental autoimmune encephalomyelitis [68]. The DMPs reported in our studies are not only consistent and validate the previous studies of gene regulatory elements in genes within the immune cell regulation cluster [14–20], but also provide an improved understanding using advanced technology as well as validation using cohorts from distant geographic locations.

It is noteworthy to mention that it is still inconclusive from previous studies or our current study, whether the significant epigenetic modifications found to associate with ME/CFS indicate a compensatory homeostatic mechanism or result from an adaptive immune response towards environmental inducers. However, these results indicate that DNA methylation constitutes a potential gene regulatory mechanism capable of mediating long-term changes in ME/CFS cases, as previously noted by other authors [18–20]. In summary, the association of differential DNA methylation with ME/CFS definitely suggests a potential role for epigenetic alterations in the pathophysiology of ME/CFS.

## 5. Conclusions

To our knowledge, this is the first study that has explored genome-wide epigenetic changes associated with ME/CFS using the new Illumina MethylationEPIC microarrays covering over 850,000 CpG sites and that has validated the results in two geographically distant cohorts of ME/CFS cases. Our findings build on previous preliminary reports showing association of altered methylation profiles of genes with immune functions and confirm DNA methylation as an epigenetic regulatory mechanism associated with ME/CFS. Future validation studies in larger cohorts of ME/CFS cases are needed to confirm these findings and evaluate the effects of such methylation patterns on gene expression.

## Supporting information

S1 Table Age and BMI matching of cases and controls in the experimental cohort from Miami/Fort Lauderdale. (DOCX)

S2 Table Age and BMI matching of cases and controls in the samples from Valencia, Spain. (DOCX)

S3 Table Global methylation values measured by ELISA-based assay. (XLSX)

S4 Table CpG sites identified as differentially methylated in ME/CFS cases compared to controls, FDR ≤ 0.05. (XLSX)

S5 Table CpG sites identified as differentially methylated in ME/CFS cases compared to controls FDR ≤ 0.1. (XLSX)

S6 Table Gene promoters identified as differentially methylated in ME/CFS cases compared to controls FDR ≤ 0.1. (XLSX)

S7 Table Gene promoters identified as differentially methylated in ME/CFS cases compared to controls FDR ≤ 0.05. (XLSX)

## References

[1] Jason LA, McManimen S, Sunnquist M, Newton JL, Strand EB. Clinical Criteria Versus a Possible Research Case Definition in Chronic Fatigue Syndrome/Myalgic Encephalomyelitis. Fatigue. 2017;5(2):89–102. 10.1080/21641846.2017.1299077 29062593 PMC5650200

[2] Brenu EW, Staines DR, Baskurt OK, Ashton KJ, Ramos SB, Christy RM, et al Immune and hemorheological changes in chronic fatigue syndrome. Journal of translational medicine. 2010;8:1 10.1186/1479-5876-8-1 20064266 PMC2829521

[3] Demitrack MA. Neuroendocrine correlates of chronic fatigue syndrome: a brief review. J Psychiatr Res. 1997;31(1):69–82. 9201649 10.1016/s0022-3956(96)00059-3

[4] Schwartz RB, Garada BM, Komaroff AL, Tice HM, Gleit M, Jolesz FA, et al Detection of intracranial abnormalities in patients with chronic fatigue syndrome: comparison of MR imaging and SPECT. AJR Am J Roentgenol. 1994;162(4):935–41. 10.2214/ajr.162.4.8141020 8141020

[5] Reyes M, Nisenbaum R, Hoaglin DC, Unger ER, Emmons C, Randall B, et al Prevalence and incidence of chronic fatigue syndrome in Wichita, Kansas. Arch Intern Med. 2003;163(13):1530–6. 10.1001/archinte.163.13.1530 12860574

[6] Jason LA, Richman JA, Rademaker AW, Jordan KM, Plioplys AV, Taylor RR, et al A community-based study of chronic fatigue syndrome. Arch Intern Med. 1999;159(18):2129–37. 10527290 10.1001/archinte.159.18.2129

[7] Lorusso L, Mikhaylova SV, Capelli E, Ferrari D, Ngonga GK, Ricevuti G. Immunological aspects of chronic fatigue syndrome. Autoimmunity reviews. 2009;8(4):287–91. 10.1016/j.autrev.2008.08.003 18801465

[8] Chandler HK, Ciccone D, MacBride RJ, Natelson B. Medically unexplained illness in short- and long-term disability applicants: prevalence and cost of salary reimbursement. Disabil Rehabil. 2008;30(16):1185–91. 10.1080/09638280701500109 17852256

[9] Reynolds KJ, Vernon SD, Bouchery E, Reeves WC. The economic impact of chronic fatigue syndrome. Cost Eff Resour Alloc. 2004;2(1):4 10.1186/1478-7547-2-4 15210053 PMC449736

[10] Jason LA, Benton MC, Valentine L, Johnson A, Torres-Harding S. The economic impact of ME/CFS: individual and societal costs. Dyn Med. 2008;7:6 10.1186/1476-5918-7-6 18397528 PMC2324078

[11] Lattie EG, Antoni MH, Fletcher MA, Czaja S, Perdomo D, Sala A, et al Beyond Myalgic Encephalomyelitis/Chronic Fatigue Syndrome (ME/CFS) Symptom Severity: Stress Management Skills are Related to Lower Illness Burden. Fatigue. 2013;1(4). 10.1080/21641846.2013.843255 PMC3837381 24278791

[12] Rowe PC, Underhill RA, Friedman KJ, Gurwitt A, Medow MS, Schwartz MS, et al Myalgic Encephalomyelitis/Chronic Fatigue Syndrome Diagnosis and Management in Young People: A Primer. Front Pediatr. 2017;5:121 10.3389/fped.2017.00121 28674681 PMC5474682

[13] Zhang X, Ho SM. Epigenetics meets endocrinology. J Mol Endocrinol. 2011;46(1):R11–32. 21322125 10.1677/jme-10-0053 PMC4071959

[14] Christopher MA, Kyle SM, Katz DJ. Neuroepigenetic mechanisms in disease. Epigenetics Chromatin. 2017;10(1):47 10.1186/s13072-017-0150-4 29037228 PMC5644115

[15] Ospelt C, Gay S, Klein K. Epigenetics in the pathogenesis of RA. Semin Immunopathol. 2017;39(4):409–19. 10.1007/s00281-017-0621-5 28324153

[16] Ciampi de Andrade D, Maschietto M, Galhardoni R, Gouveia G, Chile T, Victorino Krepischi AC, et al Epigenetics insights into chronic pain: DNA hypomethylation in fibromyalgia-a controlled pilot-study. Pain. 2017;158(8):1473–80. 10.1097/j.pain.0000000000000932 28621701

[17] Mahurkar S, Polytarchou C, Iliopoulos D, Pothoulakis C, Mayer EA, Chang L. Genome-wide DNA methylation profiling of peripheral blood mononuclear cells in irritable bowel syndrome. Neurogastroenterol Motil. 2016;28(3):410–22. 10.1111/nmo.12741 26670691 PMC4760882

[18] de Vega WC, Vernon SD, McGowan PO. DNA methylation modifications associated with chronic fatigue syndrome. PloS one. 2014;9(8):e104757 10.1371/journal.pone.0104757 25111603 PMC4128721

[19] de Vega WC, Herrera S, Vernon SD, McGowan PO. Epigenetic modifications and glucocorticoid sensitivity in Myalgic Encephalomyelitis/Chronic Fatigue Syndrome (ME/CFS). BMC medical genomics. 2017;10(1):11 10.1186/s12920-017-0248-3 28231836 PMC5324230

[20] Brenu E.W. SDR, Marshall-Gradisnik S.M. Methylation Profile of CD4+ T Cells in Chronic Fatigue Syndrome/Myalgic Encephalomyelitis. J Clin Cell Immunol. 2014;5:228.

[21] Vangeel E, Van Den Eede F, Hompes T, Izzi B, Del Favero J, Moorkens G, et al Chronic Fatigue Syndrome and DNA Hypomethylation of the Glucocorticoid Receptor Gene Promoter 1F Region: Associations With HPA Axis Hypofunction and Childhood Trauma. Psychosomatic medicine. 2015;77(8):853–62. 10.1097/PSY.0000000000000224 26230484

[22] Falkenberg VR, Whistler T, Murray JR, Unger ER, Rajeevan MS. Acute psychosocial stress-mediated changes in the expression and methylation of perforin in chronic fatigue syndrome. Genet Epigenet. 2013;5:1–9. 10.4137/GEG.S10944 25512702 PMC4222335

[23] Pidsley R, Zotenko E, Peters TJ, Lawrence MG, Risbridger GP, Molloy P, et al Critical evaluation of the Illumina MethylationEPIC BeadChip microarray for whole-genome DNA methylation profiling. Genome biology. 2016;17(1):208 10.1186/s13059-016-1066-1 27717381 PMC5055731

[24] Fukuda K, Straus SE, Hickie I, Sharpe MC, Dobbins JG, Komaroff A. The chronic fatigue syndrome: a comprehensive approach to its definition and study. International Chronic Fatigue Syndrome Study Group. Annals of internal medicine. 1994;121(12):953–9. 7978722 10.7326/0003-4819-121-12-199412150-00009

[25] Carruthers BM, van de Sande MI, De Meirleir KL, Klimas NG, Broderick G, Mitchell T, et al Myalgic encephalomyelitis: International Consensus Criteria. Journal of internal medicine. 2011;270(4):327–38. 10.1111/j.1365-2796.2011.02428.x 21777306 PMC3427890

[26] Reeves WC, Lloyd A, Vernon SD, Klimas N, Jason LA, Bleijenberg G, et al Identification of ambiguities in the 1994 chronic fatigue syndrome research case definition and recommendations for resolution. BMC health services research. 2003;3(1):25 10.1186/1472-6963-3-25 14702202 PMC317472

[27] Semler G, Wittchen HU, Joschke K, Zaudig M, von Geiso T, Kaiser S, et al Test-retest reliability of a standardized psychiatric interview (DIS/CIDI). Eur Arch Psychiatry Neurol Sci. 1987;236(4):214–22. 3582430 10.1007/BF00383851

[28] Smets EM, Garssen B, Bonke B, De Haes JC. The Multidimensional Fatigue Inventory (MFI) psychometric qualities of an instrument to assess fatigue. J Psychosom Res. 1995;39(3):315–25. 7636775 10.1016/0022-3999(94)00125-o

[29] Ware JE Jr., Sherbourne CD. The MOS 36-item short-form health survey (SF-36). I. Conceptual framework and item selection. Med Care. 1992;30(6):473–83. 1593914

[30] Krupp LB, LaRocca NG, Muir-Nash J, Steinberg AD. The fatigue severity scale. Application to patients with multiple sclerosis and systemic lupus erythematosus. Archives of neurology. 1989;46(10):1121–3. 2803071 10.1001/archneur.1989.00520460115022

[31] Bergner M, Bobbitt RA, Carter WB, Gilson BS. The Sickness Impact Profile: development and final revision of a health status measure. Med Care. 1981;19(8):787–805. 7278416 10.1097/00005650-198108000-00001

[32] Trivedi MS, Shah JS, Al-Mughairy S, Hodgson NW, Simms B, Trooskens GA, et al Food-derived opioid peptides inhibit cysteine uptake with redox and epigenetic consequences. J Nutr Biochem. 2014;25(10):1011–8. 10.1016/j.jnutbio.2014.05.004 25018147 PMC4157943

[33] Trivedi M, Shah J, Hodgson N, Byun HM, Deth R. Morphine induces redox-based changes in global DNA methylation and retrotransposon transcription by inhibition of excitatory amino acid transporter type 3-mediated cysteine uptake. Mol Pharmacol. 2014;85(5):747–57. 10.1124/mol.114.091728 24569088 PMC3990020

[34] Moran S, Arribas C, Esteller M. Validation of a DNA methylation microarray for 850,000 CpG sites of the human genome enriched in enhancer sequences. Epigenomics. 2016;8(3):389–99. 10.2217/epi.15.114 26673039 PMC4864062

[35] Assenov Y, Muller F, Lutsik P, Walter J, Lengauer T, Bock C. Comprehensive analysis of DNA methylation data with RnBeads. Nat Methods. 2014;11(11):1138–40. 10.1038/nmeth.3115 25262207 PMC4216143

[36] Teschendorff AE, Marabita F, Lechner M, Bartlett T, Tegner J, Gomez-Cabrero D, et al A beta-mixture quantile normalization method for correcting probe design bias in Illumina Infinium 450 k DNA methylation data. Bioinformatics. 2013;29(2):189–96. 10.1093/bioinformatics/bts680 23175756 PMC3546795

[37] Law CW, Alhamdoosh M, Su S, Smyth GK, Ritchie ME. RNA-seq analysis is easy as 1-2-3 with limma, Glimma and edgeR. F1000Res. 2016;5:1408 10.12688/f1000research.9005.1 27441086 PMC4937821

[38] Benjamini Y, Hochberg Y. Controlling the False Discovery Rate—a Practical and Powerful Approach to Multiple Testing. J Roy Stat Soc B Met. 1995;57(1):289–300.

[39] Zarubin T, Han J. Activation and signaling of the p38 MAP kinase pathway. Cell Res. 2005;15(1):11–8. 10.1038/sj.cr.7290257 15686620

[40] Onishi RM, Gaffen SL. Interleukin-17 and its target genes: mechanisms of interleukin-17 function in disease. Immunology. 2010;129(3):311–21. 10.1111/j.1365-2567.2009.03240.x 20409152 PMC2826676

[41] Schett G, Dayer JM, Manger B. Interleukin-1 function and role in rheumatic disease. Nat Rev Rheumatol. 2016;12(1):14–24. 10.1038/nrrheum.2016.166 26656658

[42] Egan DF, Shackelford DB, Mihaylova MM, Gelino S, Kohnz RA, Mair W, et al Phosphorylation of ULK1 (hATG1) by AMP-activated protein kinase connects energy sensing to mitophagy. Science. 2011;331(6016):456–61. 10.1126/science.1196371 21205641 PMC3030664

[43] Weil AF, Ghosh D, Zhou Y, Seiple L, McMahon MA, Spivak AM, et al Uracil DNA glycosylase initiates degradation of HIV-1 cDNA containing misincorporated dUTP and prevents viral integration. Proceedings of the National Academy of Sciences of the United States of America. 2013;110(6):E448–57. 10.1073/pnas.1219702110 23341616 PMC3568341

[44] Castelein H, Declercq PE, Baes M. DNA binding preferences of PPAR alpha/RXR alpha heterodimers. Biochem Biophys Res Commun. 1997;233(1):91–5. 10.1006/bbrc.1997.6395 9144402

[45] Zandi S, Ahsberg J, Tsapogas P, Stjernberg J, Qian H, Sigvardsson M. Single-cell analysis of early B-lymphocyte development suggests independent regulation of lineage specification and commitment in vivo. Proceedings of the National Academy of Sciences of the United States of America. 2012;109(39):15871–6. 10.1073/pnas.1210144109 23019372 PMC3465423

[46] Hardie DL, Baldwin MJ, Naylor A, Haworth OJ, Hou TZ, Lax S, et al The stromal cell antigen CD248 (endosialin) is expressed on naive CD8+ human T cells and regulates proliferation. Immunology. 2011;133(3):288–95. 10.1111/j.1365-2567.2011.03437.x 21466550 PMC3108880

[47] Stepanov GA, Filippova JA, Komissarov AB, Kuligina EV, Richter VA, Semenov DV. Regulatory role of small nucleolar RNAs in human diseases. Biomed Res Int. 2015;2015:206849 10.1155/2015/206849 26060813 PMC4427830

[48] An Y, Furber KL, Ji S. Pseudogenes regulate parental gene expression via ceRNA network. J Cell Mol Med. 2017;21(1):185–92. 10.1111/jcmm.12952 27561207 PMC5192809

[49] Marchese FP, Raimondi I, Huarte M. The multidimensional mechanisms of long noncoding RNA function. Genome biology. 2017;18(1):206 10.1186/s13059-017-1348-2 29084573 PMC5663108

[50] Adegbola A, Musante L, Callewaert B, Maciel P, Hu H, Isidor B, et al Redefining the MED13L syndrome. Eur J Hum Genet. 2015;23(10):1308–17. 10.1038/ejhg.2015.26 25758992 PMC4592099

[51] Kerr JR, Petty R, Burke B, Gough J, Fear D, Sinclair LI, et al Gene expression subtypes in patients with chronic fatigue syndrome/myalgic encephalomyelitis. The Journal of infectious diseases. 2008;197(8):1171–84. 10.1086/533453 18462164

[52] Aspler AL, Bolshin C, Vernon SD, Broderick G. Evidence of inflammatory immune signaling in chronic fatigue syndrome: A pilot study of gene expression in peripheral blood. Behav Brain Funct. 2008;4:44 10.1186/1744-9081-4-44 18822143 PMC2569951

[53] Presson AP, Sobel EM, Papp JC, Suarez CJ, Whistler T, Rajeevan MS, et al Integrated weighted gene co-expression network analysis with an application to chronic fatigue syndrome. BMC Syst Biol. 2008;2:95 10.1186/1752-0509-2-95 18986552 PMC2625353

[54] Kaushik N, Fear D, Richards SC, McDermott CR, Nuwaysir EF, Kellam P, et al Gene expression in peripheral blood mononuclear cells from patients with chronic fatigue syndrome. Journal of clinical pathology. 2005;58(8):826–32. 10.1136/jcp.2005.025718 16049284 PMC1770875

[55] Powell R, Ren J, Lewith G, Barclay W, Holgate S, Almond J. Identification of novel expressed sequences, up-regulated in the leucocytes of chronic fatigue syndrome patients. Clin Exp Allergy. 2003;33(10):1450–6. 14519154 10.1046/j.1365-2222.2003.01745.x

[56] Vernon SD, Unger ER, Dimulescu IM, Rajeevan M, Reeves WC. Utility of the blood for gene expression profiling and biomarker discovery in chronic fatigue syndrome. Dis Markers. 2002;18(4):193–9. 10.1155/2002/892374 12590173 PMC3851413

[57] Broderick G, Fuite J, Kreitz A, Vernon SD, Klimas N, Fletcher MA. A formal analysis of cytokine networks in chronic fatigue syndrome. Brain, behavior, and immunity. 2010;24(7):1209–17. 10.1016/j.bbi.2010.04.012 20447453 PMC2939140

[58] Fluge O, Bruland O, Risa K, Storstein A, Kristoffersen EK, Sapkota D, et al Benefit from B-lymphocyte depletion using the anti-CD20 antibody rituximab in chronic fatigue syndrome. A double-blind and placebo-controlled study. PloS one. 2011;6(10):e26358 10.1371/journal.pone.0026358 22039471 PMC3198463

[59] Fluge O, Risa K, Lunde S, Alme K, Rekeland IG, Sapkota D, et al B-Lymphocyte Depletion in Myalgic Encephalopathy/ Chronic Fatigue Syndrome. An Open-Label Phase II Study with Rituximab Maintenance Treatment. PloS one. 2015;10(7):e0129898 10.1371/journal.pone.0129898 26132314 PMC4488509

[60] Patarca R, Klimas NG, Lugtendorf S, Antoni M, Fletcher MA. Dysregulated expression of tumor necrosis factor in chronic fatigue syndrome: interrelations with cellular sources and patterns of soluble immune mediator expression. Clin Infect Dis. 1994;18 Suppl 1:S147–53. 8148443 10.1093/clinids/18.supplement_1.s147

[61] Liu X, Zhan Z, Xu L, Ma F, Li D, Guo Z, et al MicroRNA-148/152 impair innate response and antigen presentation of TLR-triggered dendritic cells by targeting CaMKIIalpha. Journal of immunology. 2010;185(12):7244–51. 10.4049/jimmunol.1001573 21068402

[62] Pan W, Zhu S, Yuan M, Cui H, Wang L, Luo X, et al MicroRNA-21 and microRNA-148a contribute to DNA hypomethylation in lupus CD4+ T cells by directly and indirectly targeting DNA methyltransferase 1. Journal of immunology. 2010;184(12):6773–81. 10.4049/jimmunol.0904060 20483747

[63] Brenu EW, Ashton KJ, van Driel M, Staines DR, Peterson D, Atkinson GM, et al Cytotoxic lymphocyte microRNAs as prospective biomarkers for Chronic Fatigue Syndrome/Myalgic Encephalomyelitis. J Affect Disord. 2012;141(2–3):261–9. 10.1016/j.jad.2012.03.037 22572093

[64] Galicia JC, Naqvi AR, Ko CC, Nares S, Khan AA. MiRNA-181a regulates Toll-like receptor agonist-induced inflammatory response in human fibroblasts. Genes Immun. 2014;15(5):333–7. 10.1038/gene.2014.24 24848932 PMC4111836

[65] Gambuzza ME, Salmeri FM, Soraci L, Soraci G, Sofo V, Marino S, et al The Role of Toll-Like Receptors in Chronic Fatigue Syndrome/Myalgic Encephalomyelitis: A New Promising Therapeutic Approach? CNS Neurol Disord Drug Targets. 2015;14(7):903–14. 25808894 10.2174/1871527314666150325235247

[66] Cheekatla SS, Tripathi D, Venkatasubramanian S, Paidipally P, Welch E, Tvinnereim AR, et al IL-21 Receptor Signaling Is Essential for Optimal CD4(+) T Cell Function and Control of Mycobacterium tuberculosis Infection in Mice. Journal of immunology. 2017;199(8):2815–22. 10.4049/jimmunol.1601231 PMC5636679 28855309

[67] Spolski R, Leonard WJ. Interleukin-21: a double-edged sword with therapeutic potential. Nat Rev Drug Discov. 2014;13(5):379–95. 10.1038/nrd4296 24751819

[68] Lee Y, Mitsdoerffer M, Xiao S, Gu G, Sobel RA, Kuchroo VK. IL-21R signaling is critical for induction of spontaneous experimental autoimmune encephalomyelitis. J Clin Invest. 2015;125(11):4011–20. 10.1172/JCI75933 26413871 PMC4639966

