# Supplementary material for: Identification of Myalgic Encephalomyelitis/Chronic Fatigue Syndrome-associated DNA methylation patterns
Source: PLoS One. 2018 Jul 23;13(7):e0201066. doi: 10.1371/journal.pone.0201066 (PMC6056050; doi:10.1371/journal.pone.0201066)
Supplement: S1 Table — (DOCX) [file pone.0201066.s001.docx]

**S1 Table. Demographic information and SF-36 results for ME/CFS patients and HC subjects that participated in DNA methylation analysis and validation from Florida.** * - p<0.05, Student’s t-test, ME/CFS versus HC subjects. Data are shown as mean ± standard error of mean.

|  |  | **ME/CFS Patients** | **Healthy Controls** |
| --- | --- | --- | --- |
|  | **Age (years)** | **46.3 ± 1.87** | **45.6 ± 2.31** |
|  | **BMI (kg/m^2^)** | **26.7 ± 1.38** | **25.4 ± 1.10** |
| **Physical Health** | |  |  |
|  | **Physical Functioning** | **39.7 ± 5.08*** | **97.4 ± 1.29** |
|  | **Role Physical** | **16.7 ± 9.02*** | **88.2 ± 7.25** |
|  | **Bodily Pain** | **38.3 ± 6.56*** | **87.1 ± 4.28** |
|  | **General Health** | **30.7 ± 4.65*** | **73.9 ± 5.47** |
| **Mental Health** | |  |  |
|  | **Vitality** | **26.3 ± 4.94*** | **63.7 ± 5.49** |
|  | **Social Functioning** | **34.2 ± 5.52*** | **88.8 ± 3.69** |
|  | **Role Emotional** | **60.0 ± 11.36*** | **87.7 ± 6.84** |
|  | **Mental Health** | **65.1 ± 5.73*** | **75.8 ± 5.21** |
