# Supplementary material for: Identification of Myalgic Encephalomyelitis/Chronic Fatigue Syndrome-associated DNA methylation patterns
Source: PLoS One. 2018 Jul 23;13(7):e0201066. doi: 10.1371/journal.pone.0201066 (PMC6056050; doi:10.1371/journal.pone.0201066)
Supplement: S2 Table — (DOCX) [file pone.0201066.s002.docx]

**S2 Table. Demographic information and SF-36 results for ME/CFS patients and HC subjects that participated in DNA methylation analysis and validation from Valencia, Spain.** * - p<0.05, Student’s t-test, ME/CFS versus HC subjects. Data are shown as mean ± standard error of mean.

|  |  | **ME/CFS Patients** | **Healthy Controls** |
| --- | --- | --- | --- |
|  | **Age (years)** | **54.3 ± 2.05** | **52.0 ± 1.37** |
|  | **BMI (kg/m^2^)** | **24.8 ± 1.06** | **25.8 ± 1.31** |
| **Physical Health** | |  |  |
|  | **Physical Functioning** | **51.7 ± 6.45*** | **95.9 ± 1.51** |
|  | **Role Physical** | **27.8 ± 12.73*** | **97.7 ± 7.75** |
|  | **Bodily Pain** | **33.9 ± 6.32*** | **92.5 ± 5.15** |
|  | **General Health** | **32.8 ± 3.15*** | **80.0 ± 5.87** |
| **Mental Health** | |  |  |
|  | **Vitality** | **28.7 ± 6.96*** | **84.1 ± 6.50** |
|  | **Social Functioning** | **46.7 ± 10.26*** | **98.0 ± 4.23** |
|  | **Role Emotional** | **48.1 ± 11.89*** | **87.9 ± 8.94** |
|  | **Mental Health** | **53.3 ± 7.35*** | **86.2 ± 5.82** |
